# Regional Variation in Contractile Patterns and Muscle Activity in Infant Pig Feeding

**DOI:** 10.1093/iob/obac046

**Published:** 2022-11-07

**Authors:** C J Mayerl, K A Adjerid, C E Edmonds, F D H Gould, M L Johnson, K E Steer, L E Bond, R Z German

**Affiliations:** Department of Anatomy and Neurobiology, Northeast Ohio Medical University, Rootstown, OH, 44272, USA; Department of Biological Sciences, Northern Arizona University, Flagstaff, AZ, 86011, USA; Department of Anatomy and Neurobiology, Northeast Ohio Medical University, Rootstown, OH, 44272, USA; Department of Biomedical Engineering, Tulane University, New Orleans, LA, 70118, USA; Department of Anatomy and Neurobiology, Northeast Ohio Medical University, Rootstown, OH, 44272, USA; Rowan University School of Osteopathic Medicine, Stratford, NJ, 08084, USA; Department of Anatomy and Neurobiology, Northeast Ohio Medical University, Rootstown, OH, 44272, USA; Department of Anatomy and Neurobiology, Northeast Ohio Medical University, Rootstown, OH, 44272, USA; Department of Anatomy and Neurobiology, Northeast Ohio Medical University, Rootstown, OH, 44272, USA; Department of Anatomy and Neurobiology, Northeast Ohio Medical University, Rootstown, OH, 44272, USA

## Abstract

At the level of the whole muscle, contractile patterns during activity are a critical and necessary source of variation in function. Understanding if a muscle is actively lengthening, shorting, or remaining isometric has implications for how it is working to power a given behavior. When feeding, the muscles associated with the tongue, jaws, pharynx, and hyoid act together to transport food through the oral cavity and into the esophagus. These muscles have highly coordinated firing patterns, yet also exhibit high levels of regional heterogeneity in both their timing of activity and their contractile characteristics when active. These high levels of variation make investigations into function challenging, especially in systems where muscles power multiple behaviors. We used infant pigs as a model system to systematically evaluate variation in muscle firing patterns in two muscles (mylohyoid and genioglossus) during two activities (sucking and swallowing). We also evaluated the contractile characteristics of mylohyoid during activity in the anterior and posterior regions of the muscle. We found that the posterior regions of both muscles had different patterns of activity during sucking versus swallowing, whereas the anterior regions of the muscles did not. Furthermore, the anterior portion of mylohyoid exhibited concentric contractions when active during sucking, whereas the posterior portion was isometric during sucking and swallowing. This difference suggests that the anterior portion of mylohyoid in infant pigs is functioning in concert with the tongue and jaws to generate suction, whereas the posterior portion is likely acting as a hyoid stabilizer during sucking and swallowing. Our results demonstrate the need to evaluate both the contractile characteristics and activity patterns of a muscle in order to understand its function, especially in cases where there is potential for variation in either factor within a single muscle.

## Introduction

Muscles function to power most vertebrate behaviors. The function of any given muscle, or suite of muscles, can be influenced through a variety of factors, including the timing of its activity, its lever arm, fiber properties, or contractile characteristics (Maynard Smith and Savage 1956; Biewener 1989; Biewener and Gillis 1999; Roberts et al. 2019). At the whole-muscle level, one primary source of variation in function lies in evaluating how a muscle is contracting during its activity, and whether it is actively shortening, remaining isometric, or actively lengthening (Askew and Marsh 2001; Roberts and Gabaldón 2008; Mayerl et al. 2021a). However, we have a limited understanding of the potential for variation in these characteristics within a muscle across multiple behaviors, and thus are limited in drawing conclusions about muscle function across behaviors. This is especially true for those behaviors that are not readily visible to the naked eye, such as feeding in mammals, which mostly occurs within a hidden oral cavity.

During feeding, the muscles associated with the tongue, jaws, and hyoid act in concert to manipulate and process food as it enters the oral cavity and is swallowed (Hiiemae and Crompton 1985; Hiiemae and Palmer 1999). These structures are highly coordinated to enable safe and successful oropharyngeal processing, and their activity and contractile characteristics vary on evolutionary and ontogenetic scales (Iriki et al. 1988; Westneat and Hall 1992; Thexton et al. 1998; Hylander et al. 2005; Campbell-Malone et al. 2011). In general, we have a well-developed picture of when muscles are firing during feeding and how that varies with overall food, tongue and jaw movements (German et al. 1989; German and Franks 1991; Ross et al. 2007; Orsbon et al. 2018; Feilich et al. 2021). Following oral processing, the muscles associated with the tongue and hyoid also have been demonstrated to generally fire in an anteroposterior wave during swallowing (Thexton et al. 2007), which is suspected to be correlated with moving a bolus of food posteriorly from the oropharynx into the esophagus.

Although we have a general understanding of how muscles are active during feeding, there is substantial variation in their functions. For example, many muscles exhibit different contractile characteristics during activity for different behaviors (Mayerl et al. 2021a). Furthermore, many muscles exhibit variation in their activity or contractile patterns within a single behavior (Herring et al. 1979, 1991; Konow et al. 2010; Holman et al. 2012; Mayerl et al. 2021a). During mastication in adults, the temporalis and masseter both exhibit regional heterogeneity in muscle activity and function (Herring et al. 1979, 1991) This is also true in the hyolingual muscles. For example, during infant feeding, more dorsally placed electrodes in stylohyoid record muscle activity during only swallowing, while more ventral placement reveals activity during both sucking and swallowing (Mayerl et al. 2021b). Geniohyoid and sternohyoid both show regional variation in contractile characteristics during infant feeding in pigs (Konow et al. 2010; Holman et al. 2012), and in adult macaques, the two bellies of the digastric exhibit variable contractile patterns during activity (Orsbon et al. 2020). These data suggest that the function of much of the musculature active during feeding is highly variable and depends on both the behavior being examined, as well as the specific motor units an electrode is recording from within a given muscle. It is therefore essential that care is taken to determine how variation in the anatomical location of an electrode reflects variation in how it measures both the activity of the muscle, and the muscles contractile characteristics when active.

Understanding the variation in function found in the feeding musculature can be challenging for several reasons. Many of the behaviors in question are hidden and can only be observed through technologies such as high-speed videofluroscopy or ultrasound studies (Brainerd et al. 2010; Ohkubo and Scobbie 2018). Additionally, feeding in mammals is complex from both neuromotor (Miller 1999; Jean 2001; Mistry and Hamdy 2008) and mechanical perspectives (Hiiemae and Crompton 1985). When feeding, mammals go through a series of feedback loops from ingestion, to transport and mastication, to eventually swallowing (Hiiemae and Crompton 1985). How those feedback loops interact can depend on a variety of factors such as food mechanical properties and bite location (Agrawal et al. 1998; Vinyard et al. 2008).

Unlike adults, which exhibit complex oral processing behaviors, infant mammals feed on only one food source (milk), so variation in food properties play a minimal role in feeding, and the complex process model of feeding is reduced two three primary stages that are generally linear: (1) ingestion, (2) transport, and (3) swallowing (German et al. 1992; Mayerl et al. 2020a). Infants thus represent a valuable simplified model for studying the interaction between muscle function and feeding behavior. We used an infant mammal to explicitly investigate the role of regional heterogeneity in the function of two muscles associated with feeding: mylohyoid and genioglossus. Both of these muscles have been previously identified to show regional heterogeneity in firing patterns (German et al. 2009; Thexton et al. 2012), although regional heterogeneity has not been systematically evaluated on an anteroposterior axis. Furthermore, our recent work on the hyolingual muscular anatomy illustrated a potential mechanism to explain regional heterogeneity in function in infant pigs, as we observed two distinct portions of mylohyoid through contrast-enhanced microCT scans. Specifically, we were interested in the following questions: (1) Are there differences in muscle firing patterns associated with sucking and swallowing in anterior vs. posterior parts of mylohyoid and genioglossus? (2) Are there differences in contractile patterns between anterior and posterior parts of mylohyoid during sucking? As geniohyoid is not a strap-shaped muscle with linear fibers, understanding its contractile patterns is outside the scope of this manuscript, and warrants future study.

These results provide important insights into the function of the feeding musculature in mammals and highlight the need to understand a muscles activity patterns and contractile characteristics to interpret muscle function. Furthermore, they highlight the importance of evaluating variation in function within a muscle.

## Methods

### Animal housing and care

Care for infant pigs followed standard procedures (Mayerl et al. 2019, 2020b, 2021c). Four 2-day-old pigs (*Sus scrofa* Linnaeus 1758) were obtained from a commercial farm (Yorkshire/Landrace Cross, Shoup Investments, Orville, OH, USA). Infant pigs were trained to drink infant pig formula (Solustart Pig Milk Replacement, Land O'Lakes, Arden Mills, MN, USA) from a bottle and commercially available nipple (NASCO Farm & Ranch, Fort Atkinson, WI, USA). All animal care and procedures were approved by the Institutional Animal Care and Use Committee at NEOMED (Protocol #19-03-222).

### Anatomical data collection

Anatomical data used in this study were collected from a specimen used in Mayerl et al. (2021a, 2021b). In short, the specimen (21 days of age at time of death) was stained initially by perfusion with a 5% I2Ki solution through the common carotid to reduce staining time. Following perfusion, the pig was fixed in formalin (10%) for 14 days, and then placed in a 2.5% I2Ki solution refreshed every 14 days until complete and even staining occurred (approximately 1 month total). Following this, the pig was scanned using a micro CT scanner (35 µm, 70kVp, 22 µA) following standard DiceCT protocols (Gignac et al. 2016).

**Fig. 1:**
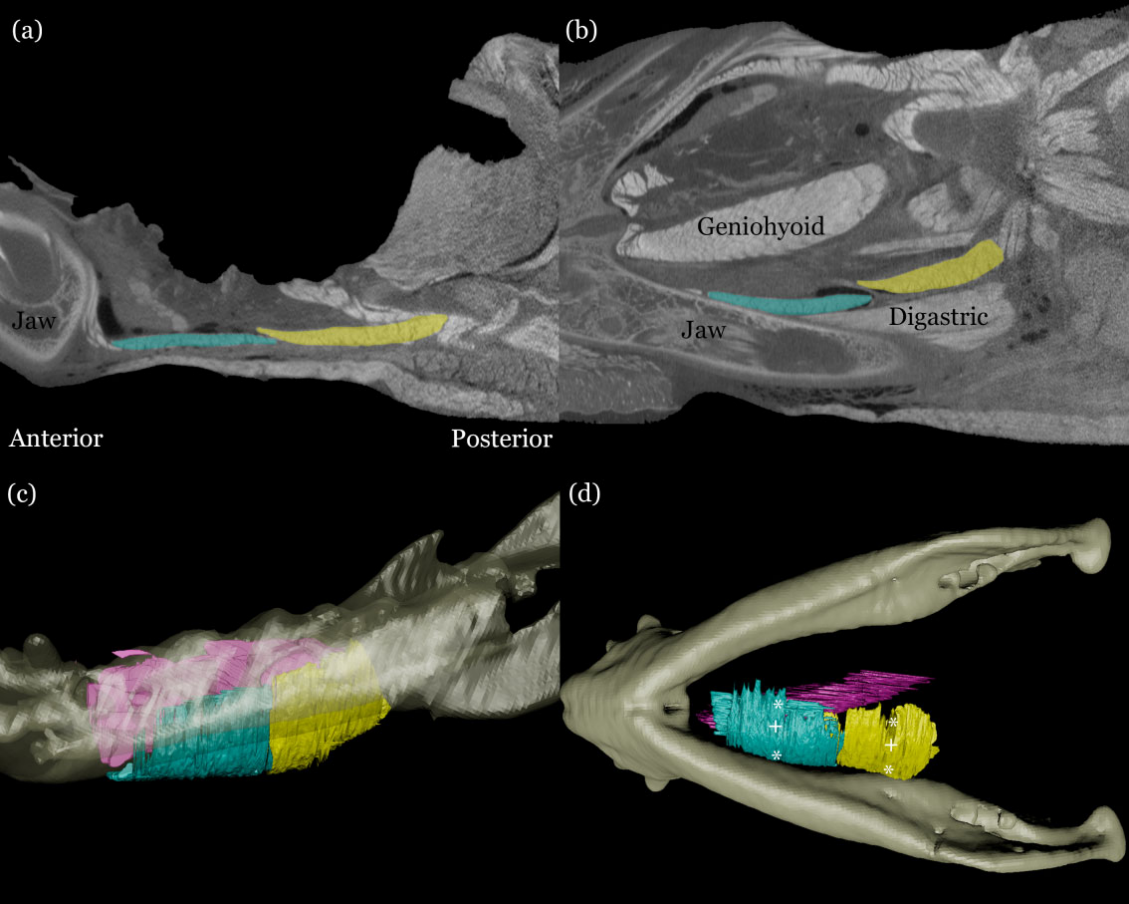
Contrast-enhanced CT scan with an anteroposterior slice (a, left is anterior), dorsoventral slice (b, bottom is ventral), and lateral (c, left is anterior) and ventral (d) three dimensional renders of anterior mylohyoid (blue), posterior mylohyoid (Yellow) and genioglossus (pink). The symbol * in D represents locations of bead placement for fluoromicrometry; + and ^ in D represent placement of electrodes for EMG in mylohyoid and genioglossus respectively. Labels in (a) and (b) are provided for reference.

Whole-muscle anatomical data were segmented using Avizo 9.4 (FEI Visualizations Science Group, Hillsboro, OR, USA). Endomysium and muscle fascicles were identified as high-density material surrounded by lower-density material (perimysium) below the minimum gray-scale threshold included in segmented volumes. We identified and isolated two distinct bellies of mylohyoid, the first documentation of this in pigs, as well as the genioglossus muscle (Fig. 1, Movie 1). Muscles were exported as .obj files and were imported into Autodesk Maya (2020; Autodesk, Inc., San Rafael, CA, USA) along with models of the jaw for visualization. DiceCT data were collected and processed prior to surgical procedures, and used to identify and guide the insertion of EMG wires and radiopaque markers.

### Surgical procedures

Pigs underwent three separate procedures prior to data collection. (1) Tantalum markers were implanted under isoflurane anesthesia into homologous locations across animals on the hard palate, midline of the tongue (anterior, middle, and posterior locations), soft palate, palatopharyngeal arches, and subdermal space dorsal to the snout at 4 days of age (not discussed here). (2) At between five and 6 days of age, pigs underwent a sterile surgery in which tantalum beads were sutured to the fascia over the thyroid eminence, and between the location of the insertion of the two bellies of sternohyoid to approximate hyoid translations during X-ray data recording (not discussed in this paper). (3) From ages 18–20 days, pigs underwent a sterile surgery for the implantation of fluoromicrometry beads and electrodes to measure muscle activity (the focus of this paper).

In this surgery, bipolar individually insulated wires were injected into genioglossus in anterior and posterior locations, as well as into the left and right bellies of thyrohyoid using a 19-gauge needle (Fig. 1). Electrodes were placed in thyrohyoid due to its stereotyped firing pattern occurring only during swallows. Genioglossus was identified by parting the two bellies of mylohyoid near the midline with blunt dissection and no cutting or tearing of the muscle and then parting the two bellies of geniohyoid. Electrodes in the anterior position were inserted by angling the needle anteriorly through the window into the genioglossus, whereas electrodes in the posterior location were inserted by angling the needle posteriorly. Thyrohyoid was identified as the muscle lying on the ventral surface of the larynx between the thyroid eminence and the hyoid bone. As the infant pig mylohyoid muscle is fairly thin, custom made electrode patches (5 × 5 mm patches with 2 mm of exposed wire) were sewn onto the muscle in anterior and posterior locations, with exposed sections lying directly against the muscle (Fig. 1d). Prior to patch placement, the anterior and posterior bellies of mylohyoid were identified using blunt dissection We also sutured laser drilled 1.0 mm tantalum beads (X-Medics, Frederiksberg, Denmark) along the fiber direction of mylohyoid, with a bead just lateral to the midline of the animal on the left, and a second bead close to the lateral margin of the muscle. One set of beads were placed in an anterior location of the muscle, and another set of beads were sutured posteriorly.

Electrodes were sutured to connective tissue in the body to provide a means of strain relief, and left the body through the posterior area of the incision. Electrodes were pre-operatively soldered to a 6-pin microconnector, which was attached to a 25-pin D connector. Cables outside of the incision were secured with self-adhering wrap to minimize the risk of pigs damaging electrode wires, and pigs wore a custom backpack to secure the D-connector. Following data collection, animals were euthanized and electrode position was confirmed with dissection.

### Data collection

Approximately 12–36 h after surgery, we collected digitally synchronized EMG data with biplanar X-ray video. EMG data were amplified and recorded at 10 kHz (MA-300, Motion Laboratory Systems, Baton Rouge, LA, USA) on a 16-channel Powerlab (16/35, ADInstruments, Colorado Springs, CO, USA). Video data were collected in lateral and dorsoventral views at 100 fps (XC1 M, XCItex, Cambridge, MA, USA) during fluoroscopic exposure (GE 9400 C-Arm, 75kV, 5 mA) following standard XROMM undistortion and calibration of images protocols (Brainerd et al. 2010). During data collection, pigs fed *ad libitum* on milk mixed with barium (0.2 cups per cup of water) from a bottle and nipple while standing in a radiolucent box. We recorded at least 18 swallows per pig, with sucks varying by the number of sucks it took per swallow per pig (Supplementary Table S1).

### Data processing

We manually identified frames where sucks began and ended from lateral views of our X-ray video. Sucks were identified as beginning at the frame when the tongue made contact with the hard palate posterior to the end of the nipple, and ending the frame prior to the beginning of the following suck following published procedures ([Bibr bib31][Bibr bib32]). Swallows were identified as beginning on the frame where the bolus accumulated in the valleculae before passing to the epiglottis, and ending on the frame that the epiglottis returned to a resting position (Edmonds et al. 2020; [Bibr bib34][Bibr bib37]).

Fluoromicrometry data were tracked from the entire feeding sequence of identified sucks and swallows and processed using XMALab (Knörlein et al. 2016). Marker to marker precision within a rigid body was ∼0.005 cm. Kinematic data were filtered with a 10 Hz low-pass Butterworth filter to remove noise, and three dimensional intermarker distances between anterior and posterior bead pairs were exported from XMALab. These distances were processed through a custom MATLAB script that separated the feeding sequence into isolated events using our manually determined suck and swallow frames. For each set of data, we calculated raw intermarker distance during a behavior as well as intermarker distance interpolated throughout the duration of a behavior into a percentage of the total duration of a behavior (101 points). To examine contractile characteristics, we standardized intermarker distance across individuals and across electrode locations by setting the intermarker distance to 0 at the frame of muscle activity onset.

A bandpass filter was applied to EMG data to reduce noise (150 Hz low-pass, 3000 Hz high-pass), and exported from Labchart at 10 kHz. Raw data were rectified, integrated, and threshold noise levels were determined using custom code in R (version 4.0.3) based on published procedures (Thexton 1996). Within a suck, onset was determined as the time where a channel first passed the threshold noise value, and offset was determined as the last instance where a signal was generated above noise values. We calculated onset and offset in raw time, as well as determined at what time the muscle turned on or off relative to the percentage of the suck. As a burst only occurred once within a cycle, this process was the same for sucks, as well as sucks that included a swallow (Thexton et al. 2012).

### Statistical analyses

All statistical analyses were performed using R (v 4.0.3). To compare muscle firing timing, we used linear mixed effects models (lme4; Bates et al. 2015), with behavior (suck or sucks that include a swallow) and electrode location (anterior or posterior) and their interaction as fixed effects, and individual as a random effect. We calculated χ^2^ and *P*-values using the ANOVA function in R, and where an interaction was significant, performed planned contrast analyses and calculated Cohen's *D*-values from those contrasts (Cohen 1992). Due to variation in marker placement, we present contractile characteristics on a per-individual basis, and did not perform statistical analyses.

## Results

### EMG activity during sucking and swallowing

**Fig. 2:**
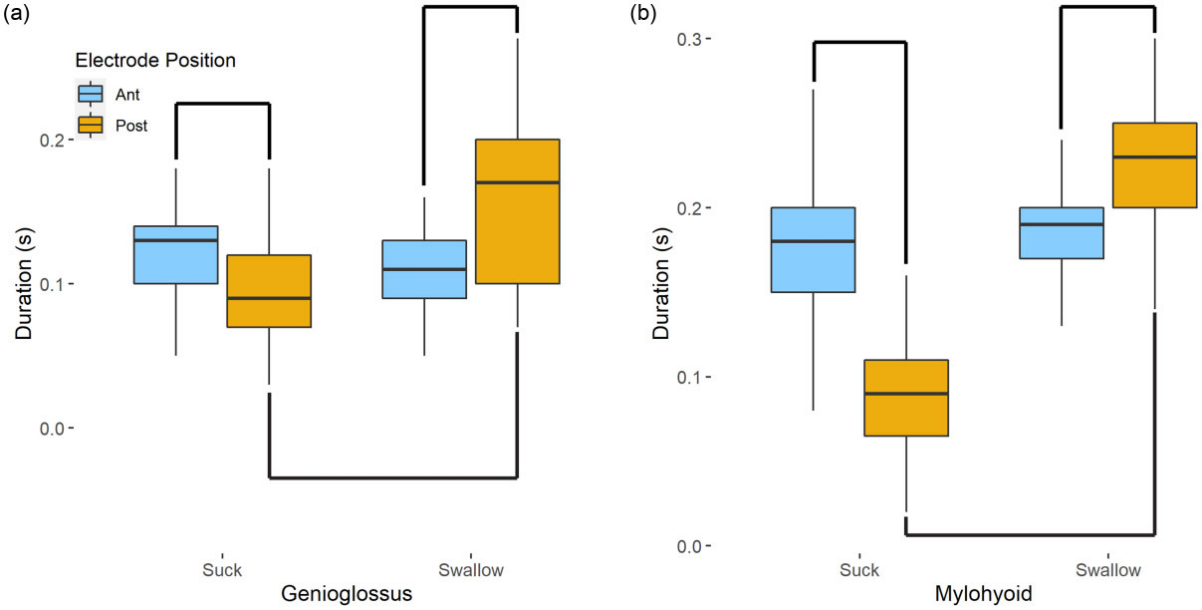
EMG duration of genioglossus (a) and mylohyoid (b) between sucking and swallowing bursts does not differ in the anterior portion of the muscle (blue) but is longer in the posterior belly during a swallow (yellow). During sucks, the posterior part of the muscle was active shorter than the anterior part for both muscles. Lines connecting plots indicate statistical significance as identified by planned contrast analyses.

Overall, we found regional variation in EMG activity of genioglossus and mylohyoid for sucking vs. swallowing, as well as differences in contractile characteristics in mylohyoid depending on electrode location within a muscle. We found that electrodes in anterior parts of genioglossus and mylohyoid did not vary in firing patterns for suck bursts or sucks that included a swallow (Fig. 2; Table 1). However, in the posterior portion of both muscles the duration of the burst was significantly longer during a suck that included a swallow than during an isolated suck (Fig. 2; Tables 1 and 2).

**Table 1.** Mixed effects models results for muscle timing across electrode location

	Ant suck (Mean ± SD)	Post suck (Mean ± SD)	Ant swallow (Mean ± SD)	Post swallow (Mean ± SD)	Suck Swallow (χ^2^, *P*)	Ant Post (χ^2^, *P*)	Interaction (χ^2^, *P*)
Genioglossus duration (s)	0.122 ± 0.03	0.093 ± 0.03	0.111 ± 0.03	0.161 ± 0.06	**119.8, <0.001**	**27.5, <0.001**	**189.9, <0.001**
Mylohyoid duration (s)	0.18 ± 0.03	0.09 ± 0.03	0.18 ± 0.04	0.23 ± 0.04	**574.9, <0.001**	**558.7, <0.001**	**446.2, <0.001**
Genioglossus Onset (s)	0.08 ± 0.06	0.12 ± 0.09	0.10 ± 0.07	0.06 ± 0.07	0.81, 0.37	**73.1, <0.001**	**113.3, <0.001**
Genioglossus Offset (s)	0.20 ± 0.07	0.21 ± 0.10	0.21 ± 0.08	0.23 ± 0.07	**49.0, <0.001**	**11.9, <0.001**	0.68 0.41
Mylohyoid Onset (s)	−0.11 ± 0.03	−0.05 ± 0.03	−0.10 ± 0.04	−0.03 ± 0.04	**19.0, <0.001**	**1840, <0.001**	**4.65, 0.031**
Mylohyoid Offset (s)	0.07 ± 0.04	0.04 ± 0.03	0.08 ± 0.04	0.20 ± 0.04	**830.3, <0.001**	1.59, 0.21	**590.9, <0.001**

Onset and offset timings are relative to the beginning of the suck cycle as identified by X-ray video. Bolded values indicate statistically significant differences between groups.

**Table 2.** Results from planned contrasts analyses for muscle timing data between electrode locations (Cohen's *D*, *P*)

	Suck ant vs. swallow ant	Suck post vs. swallow post	Suck ant vs. suck post	Swallow ant vs. swallow post
Genioglossus duration	0.38, 0.026	**−1.68, <0.001**	**0.93, <0.001**	**−1.10, <0.001**
Mylohyoid duration	−0.25, 0.05	**−4.31, <0.001**	**2.73, <0.001**	**−1.13, <0.001**
Genioglossus onset	−0.25, 0.117	0.66, <0.001	−0.52, <0.001	0.45, <0.001
Genioglossus offset	−0.07, 0.64	−0.12, 0.18	−0.11, 0.29	−0.22, 0.24
Mylohyoid onset	−0.29, 0.03	−0.48, <0.001	**−1.87, <0.001**	**−1.72, <0.001**
Mylohyoid offset	−0.42, <0.001	**−4.6, <0.001**	0.69, <0.001	**−2.66, <0.001**

Bolded values indicate statistically significant differences with large effects sizes.

**Fig. 3:**
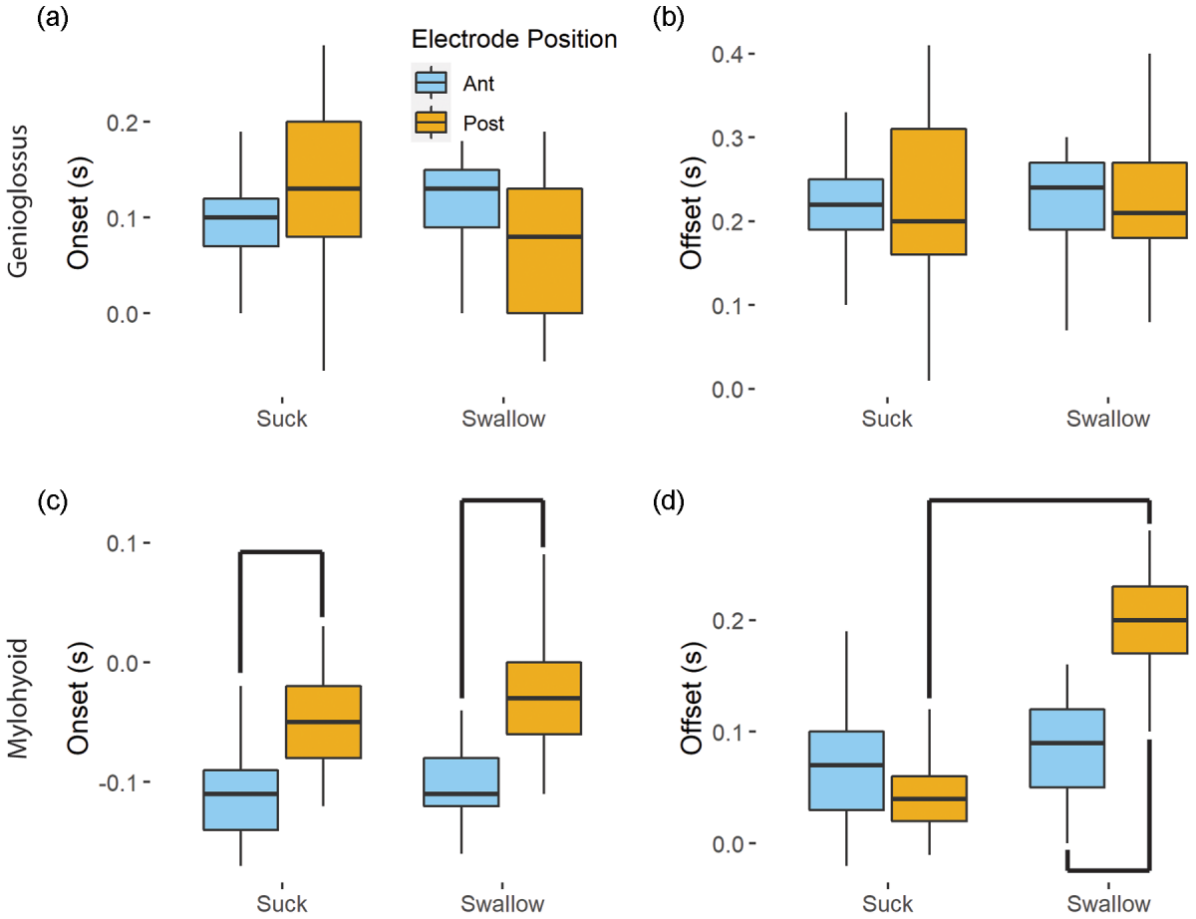
The time of muscle onset and offset relative to the beginning of the suck for genioglossus (a, b) and mylohyoid (c, d) for sucks and sucks involving a swallow in anterior (blue) and posterior (yellow) portions of the muscle. Lines indicate statistically significant differences with large effects sizes. No differences were observed in genioglossus, whereas onset timing followed an anteroposterior wave in mylohyoid (c), and offset was later in posterior electrodes during a swallow than during a suck (d).

We found no consistent differences between anterior and posterior bellies during sucking or swallowing during a suck in muscle onset or offset for genioglossus (Fig. 3a, b; Tables 1 and 2). However, we found an anteroposterior pattern of muscle onset activity in mylohyoid for sucks (Fig. 3c, Ant On: −0.11 ± 0.03s, Post on: −0.05 ± 0.03s, *P* < 0.001, *D* = −1.87), and for sucks that had a swallow (Tables 1 and 2). We also found the posterior portion of mylohyoid turned off later than the anterior portion during a suck that included a swallow (Fig. 3d; Table 2). Furthermore, we found no differences in onset or offset in the anterior portion of mylohyoid during sucking or swallowing, but that electrodes in posterior positions recorded a later time of offset during a suck that included a swallow than an isolated suck (Suck off: 0.04 ± 0.03 s, Swallow off: 0.20 ± 0.04 s, *P* < 0.001, *D* = −4.6, Table 2).

### Muscle contractile characteristics during activity

**Fig. 4:**
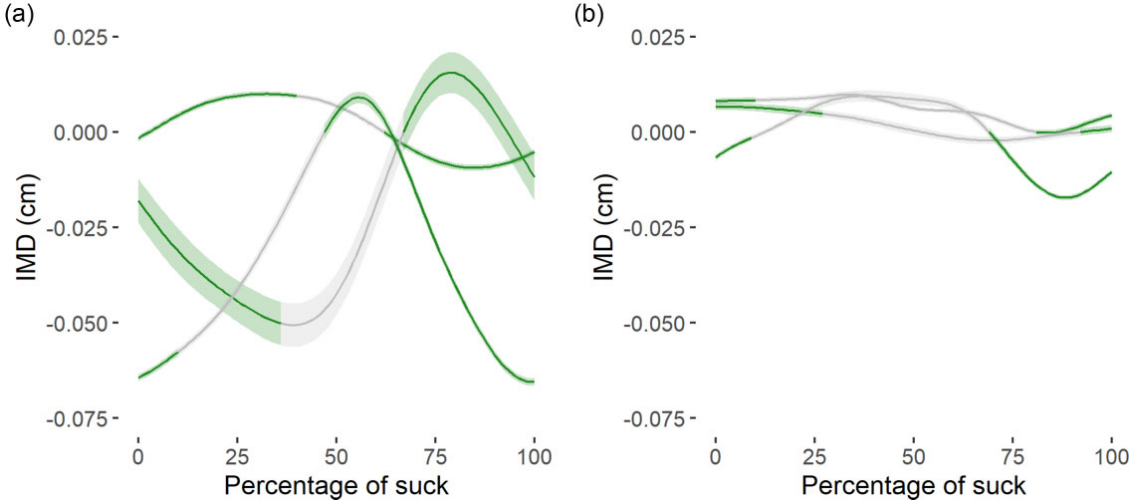
Mean length changes (solid line) ± one standard error (shaded region) in the anterior (a) and posterior (b) bellies of mylohyoid during sucking. Each line represents one individual; green: muscle is on; gray: muscle is off; IMD: Intermarker distance, standardized to be 0 at the time of muscle onset during a behavior. Note that the excursion of movement illustrated here is less than the typical excursion due to variation in when the minimum and maximum distances occurred across cycles within an individual.

**Fig. 5:**
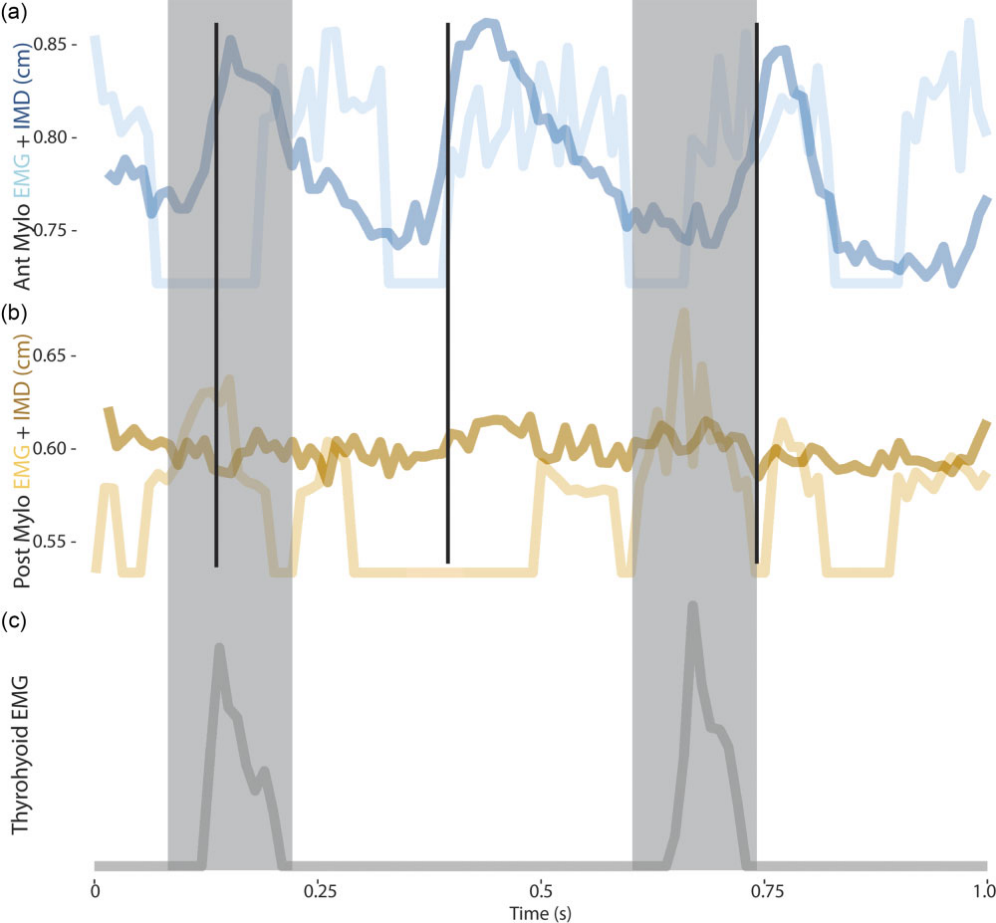
Example raw (unstandardized by onset timing) data from one pig for anterior mylohyoid EMG and IMD data (a), posterior mylohyoid EMG and IMD data (b), and thyrohyoid EMG data (c) for 1 s. Black lines indicate the beginning of a suck cycle, gray bars indicate the duration of a swallow, highlighted by thyrohyoid firing (gray) to demonstrate changes in EMG activity during posterior mylohyoid. Anterior mylohyoid does not change EMG activity during swallowing, and is primarily concentric during sucking (a), whereas posterior mylohyoid has changes in EMG activity during swallowing, but remains isometric during all activity (b). IMD; intermarker distance.

Due to either electrode or suture failure, we collected data on contractile characteristics from anterior and posterior locations from three of the four pigs (Fig. 4). The anterior portion of mylohyoid underwent substantive length changes throughout a sucking cycle (Fig. 4a; Supplementary Table S2). Activity generally began as a concentric contraction and ended close to the beginning of muscle lengthening (Fig. 4), although one individual had a slight offset in this pattern, likely due to slight variation in electrode and bead placement across individuals (Fig. 4a). In contrast, the poster portion of mylohyoid exhibited little length change during activity during sucking and was essentially isometric (Fig. 4B; Supplementary Table S2). These patterns did not change during activity when swallowing, and we chose to focus on sucking contractile characteristics here (Fig. 5, Supplementary Fig. S2).

## Discussion

Muscle function during infant feeding was heterogeneous across multiple scales. One substantial difference was between electrodes in the anterior vs. posterior portions of a muscle. Electrodes placed in anterior positions of the muscle belly in mylohyoid and genioglossus did not vary in their activity or contractile patterns (in the case of mylohyoid) during isolated sucks *vs.* sucks that included a swallow. This is most likely due to the imprecise nature of electrode placement across individuals in this muscle, and future research more explicitly implanting electrodes in genioglossus would test this possibility. In contrast, electrodes placed more posteriorly exhibited changes in activity during both sucking and sucks involving a swallow in both muscles. During sucks that included a swallow, posteriorly placed electrodes fired for a longer period of time, likely as a reflection of the increased duration of the behavior (a single suck vs. a suck and a swallow, Supplementary Fig. S1). Interestingly, this was only true for genioglossus, as posterior electrodes in mylohyoid continued to fire for longer during a suck-swallow than during a suck even after correcting for total behavioral duration (Supplementary Fig. S1). Similarly, the contractile characteristics of muscles associated with infant feeding show variation within a muscle. In mylohyoid, the anterior portion of the muscle exhibited rhythmic concentric contractions, whereas the posterior portion of the muscle was isometric throughout activity.

### Function of variation in firing patterns within a muscle

This work contributes to a growing body of literature that demonstrates that muscle firing patterns are heterogeneous within muscles associated with the hyolingual apparatus. Stylohyoid (Mayerl et al. 2021a), geniohyoid (Holman et al. 2012), sternohyoid (Konow et al. 2010), and genioglossus (Mu and Sanders 2010), as well as oropharyngeal muscles in general (German et al. 2008, 2009), have all been demonstrated to exhibit high levels of regional heterogeneity in muscle firing patterns and motor supply. This suggests that motor unit territories in much of the feeding musculature are small (Herring et al. 1991; Mu and Sanders 2010). Small motor territories are likely useful in the context of variation in oropharyngeal function, both from ontogenetic and evolutionary perspectives, as they may facilitate more fine recruitment of muscles for different functions.

There are several likely explanations for small motor units within the hyolingual musculature. One is that movements of the tongue and of the hyoid, powered by the muscles attaching to it, are essential for a variety of behaviors, including feeding, vocalization, and breathing (German et al. 2011). Similarly, in mammals the anatomy of the head and neck are complicated, and multiple functions must be possible within a small amount of anatomical space. Here, by evaluating muscle firing patterns within muscles both spatially and across behaviors, we have illustrated that some parts of the muscle change their firing patterns for different behaviors (e.g., posterior mylohyoid), while others do not. Unlike anterior portions of mylohyoid, posterior mylohyoid fibers have been previously demonstrated to have separate functional subgroups that are activated during a swallow (Meng et al. 1999; Thexton et al. 2012). We only investigated muscle function during infancy, and future work systematically evaluating how these muscles change in function through and beyond weaning could provide useful insights into how muscle firing patterns and functions have the potential to vary given different behaviors such as drinking, or chewing on foods of different properties (Westneat and Hall 1992; Cundall et al. 2012). Furthermore, our results are from a limited number of individuals, and sample from a limited region within a muscle, and there is potential for variation to exist in our specific interpretations if more individuals or sampling locations were analyzed.

Intra-muscle variation in EMG activity of single muscles during the swallow also provides more detail on the complexity of the organization of the swallowing motor pattern. The anteroposterior wave of activation of muscles during the swallow (Thexton et al. 2007) requires quasi-simultaneous activation of muscles innervated by motor neurons located in anatomically disparate brainstem nuclei (e.g., trigeminal motor nucleus and hypoglossal nucleus). This complex pattern of motor neuron recruitment is associated with a two-part swallow pattern generating circuit, where the second circuit, located in the ventral swallowing group, projects to the various motor neuron nuclei and is therefore responsible for appropriate recruitment (Jean and Dallaporta 2006). However, the anterior to posterior patterning within the mylohyoid itself during the swallow indicates that this ventral swallowing group also temporally patterns recruitment of motor neurons within motor nuclei.

### Contractile characteristics

Acquiring data on only muscle activity during a specific behavior provides an incomplete picture of the function of a muscle during that behavior. For example, within the hyolingual musculature, all muscles studied to date exhibit some combination of isometric, eccentric, and concentric contractile patterns during different activities, which results in those muscles changing in function throughout a given behavior (Orsbon et al. 2020; Mayerl et al. 2021a, 2021b) Thus, to understand muscle function, we must examine muscle activity within the context of its contractile patterns during a behavior.

We demonstrated that the anterior portion of mylohyoid was primarily concentric during activity when suckling (Fig. 4). We did observe some variation in muscle contractile characteristics in the anterior portion of mylohyoid, which is likely due to a combination of factors. These include capturing only a portion of the fascicle movements and activities, variation in exact location in electrode placement, as well as variation in how deep an infant places the nipple in their mouth, which would impact the temporal measure of when a suck would begin. However, overall, we suggest that mylohyoid, as the floor of the oral cavity, likely functions to work in concert with the jaw, genioglossus, and intrinsic tongue musculature to generate suction in the oral cavity to acquire milk (Thexton et al. 2012). In contrast, the posterior belly of mylohyoid was primarily isometric during activity, for both sucking and swallowing. This suggests that it is functioning primarily to dampen the contractions of the anterior belly of mylohyoid during suckling to stabilize the hyoid, a role that it continues to do during a swallow, where the hyoid is elevated. If posterior mylohyoid remains isometric even when the hyoid is moving, it's orientation must change as the hyoid elevates (Orsbon et al. 2020; Mayerl et al. 2021a, 2021b), which we were unable to quantify here but would be an interesting line of question for future work. These data highlight the importance of acquiring data on the activity of a muscle as well as its contractile characteristics in order to understand its function.

Beyond mylohyoid, there are several muscles that exhibit regional variation in contractile characteristics across different behaviors, and even within a single muscle. In infants, both geniohyoid and sternohyoid show different contractile patterns depending on sampling location within the muscle (Konow et al. 2010; Holman et al. 2012). Similarly, digastric shows variation in contractile patterns in adult monkeys when feeding (Orsbon et al. 2020). This variation across the hyolingual musculature likely reflects variation in changes in firing patterns and plays a role in the varied functions of these muscles across and within behaviors.

### Open questions and future directions

While this work presents an important step forward in our understanding of how the hyolingual musculature is functioning to power feeding in mammals, there is substantial room for future research. We have focused on only two muscles, in one species, and at one specific time point in development (infancy), when mammals are feeding on a relatively homogeneous food source. Furthermore, we found regional variation in EMG duration in genioglossus, and future studies that systematically evaluate genioglossus function would provide insight into its specific function during feeding behaviors. This is especially true in the context of the varying functions of the tongue, and understanding how the extrinsic tongue musculature functions during food acquisition and processing is an open question. Some research has focused on the general characteristics of shape change in the mammalian adult tongue (Liu et al. 2008; Orsbon et al. 2020; Feilich et al. 2021; Olson et al. 2021), but this has been limited to the tongue as an entire structure, and has not investigated how the different components of the tongue are controlled and functioning. There is also potential for the function of both genioglossus and mylohyoid to change within mammals ontogenetically. For example, Orsbon et al. (2020) found that mylohyoid activity was concentric during food processing in adult primates, although it is impossible to determine if these differences are reflective of changes in ontogeny or phylogeny. Even within pigs, there is the potential that the anatomy and physiology of the hyolingual muscles change with age (Mayerl et al. 2021a). Thus, one promising avenue for future research involves studying the function of the hyolingual musculature through ontogeny, as well as across different species, and even across different behaviors within a species.

The high level of variation found across the hyolingual musculature is interesting, especially considering that not all muscles display this variation. For example, thyrohyoid and cricothyroid both exhibit stereotyped firing patterns during activity in mammals (Thexton et al. 2007). One possible explanation for why many, but not all muscles associated with the hyoid have heterogeneous firing and contractile characteristics is that muscles that have evolved within mammals *de novo* may not carry the phylogenetic history of muscles present across vertebrates, such as mylohyoid (intermandibularis in other vertebrates, Konow et al. 2010). Further investigating the function of the hyolingual musculature in non-mammalian vertebrates during different behaviors could help to elucidate this possibility.

From the perspective of neural control of feeding physiology, the similarities in the anterior portions of the muscles between activation in sucking and swallowing raises further questions, especially considering that the two behaviors are thought to be under control by separate brainstem circuits. At least two possibilities could account for this pattern: one involving tight coordination between sucking and swallowing centrally (such that the swallowing group is only activated at a certain point in the cyclical activation of the sucking cycle), the other involving separate recruitment of those motor units by the swallowing cycle and inhibition of the sucking pattern generator in suck-swallow cycles. Much more work on the neural architecture of the swallowing and sucking brainstem complexes is needed to address these possibilities.

## Conclusions

Muscle function during infant feeding is complex, and interpretations of function based on activity without consideration for contractile characteristics or the potential for regional variation may not be accurate. Our results highlight that the function of both genioglossus and mylohyoid likely vary across the length of the muscle, and that that variation is additionally reflected by differences in EMG patterns during different behaviors. Thus, in order to test the role any given muscle plays in any given behavior, we must have an understanding of both the anatomical position an electrode is recording from in the muscle, as well as what the contractile characteristics of the muscle are at the electrode's location.

## Supplementary Material

obac046_Supplemental_File

## Data Availability

Data used in statistical analyses are available on Figshare at https://doi.org/10.6084/m9.figshare.21395562.
