# Supplementary material for: Regional Variation in Contractile Patterns and Muscle Activity in Infant Pig Feeding
Source: Integr Org Biol. 2022 Nov 7;4(1):obac046. doi: 10.1093/iob/obac046 (PMC9756950; doi:10.1093/iob/obac046)
Supplement: obac046_Supplemental_File [file obac046_supplemental_file.zip › Supplementary Material.pdf]

Supplementary Material:

Movie 1. Animation of the anatomy of genioglossus (pink) anterior (blue) and posterior mylohyoid (yellow) relative to the lower jaw in an infant pig.

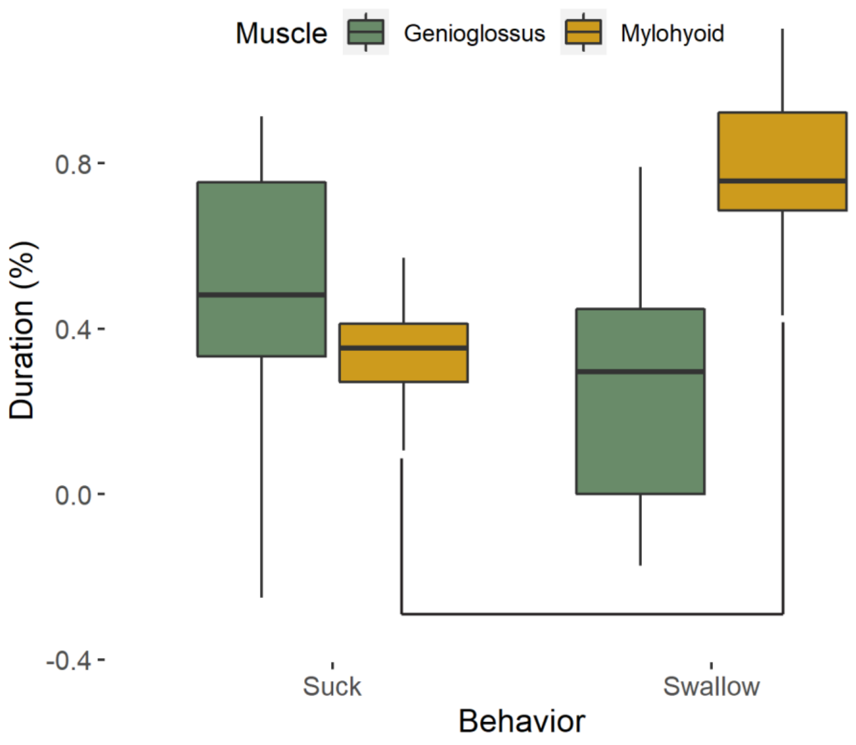

Figure S1. When standardized as a percentage of activity throughout the duration of the entire behavior (either just a suck, or a suck + swallow), the posterior portion of genioglossus (green) does not fire for longer during swallows than sucks, although mylohyoid (tan) does. Lines between box plots indicate a large effect size between two groups.

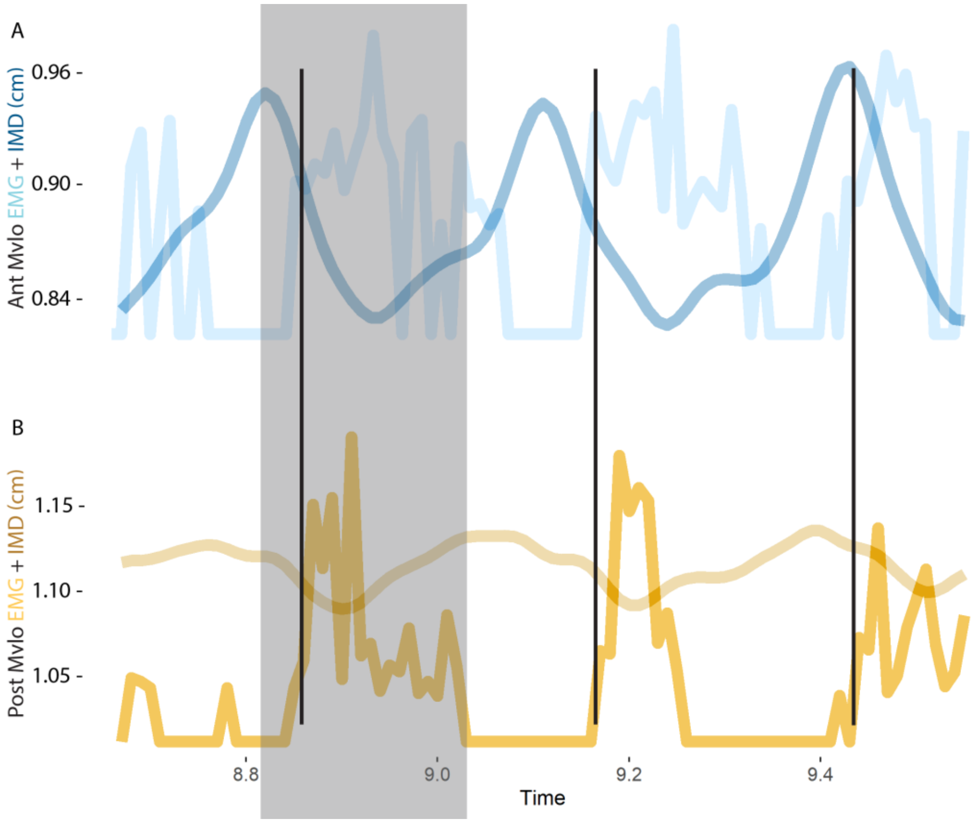

Figure S2. Example raw (unstandardized by onset timing) data from one pig for anterior mylohyoid EMG and IMD data (A) and posterior mylohyoid EMG and IMD data (B) for one second. Black lines indicate the beginning of a suck cycle, grey bars indicate the duration of a swallow, highlighted to illustrate changes in EMG activity in posterior mylohyoid. Anterior mylohyoid does not change EMG activity during swallowing, and is primarily concentric during sucking (A), whereas posterior mylohyoid has changes in EMG activity during swallowing, but has little length change throughout activity (B). IMD; Intermarker distance.

Table S1. Count of sucks and swallows used for statistical analyses of EMG activation by pig, and for calculations of intermarker distance during activity.

Pig	Sucks	Swallows
1	78	18
2	43	18
3	48	18
4	103	18

Table S2. Mean ± standard error intermarker distance excursion, maximum and minimum timing (as a percentage of the suck cycle) for anterior and posterior mylohyoid across individuals, demonstrating the low excursions and high variability in posterior mylohyoid.

	1	2	3	4
AntExc	0.022 ± 0.006		0.076 ± 0.003	0.085 ± 0.001
AntMaxTime	37.8 ± 1.71	±	75.1 ± 2.34	54.0 ± 0.57
AntMinTime	87.6 ± 1.24	±	39.7 ± 1.67	99.4 ± 0.30
PostExc	±	0.017 ± 0.001	0.015 ± 0.001	0.033± 0.001
PostMaxTime	±	61.2 ± 2.27	35.4 ± 4.34	40.4± 1.23
PostMinTime	±	44.2 ± 4.75	56.3 ± 3.74	88.4 ± 0.73

Ant: anterior mylohyoid; Post: posterior mylohyoid MaxTime: time of maximum distance apart as a percentage of the suck cycle; MinTime: time of minimum distance apart as a percentage of the suck cycle.
